# A Metastasis of Ovarian Cancer in the Bartholin Gland: A Case Report with Systematic Literature Review

**DOI:** 10.1007/s43032-023-01373-y

**Published:** 2023-10-04

**Authors:** Gregor Leonhard Olmes, Georg Peter Breitbach, Anton Tepikin, Adriana Nistor, Erich Franz Solomayer, Bashar Haj Hamoud

**Affiliations:** 1 Department of Gynecology, Obstetrics and Reproductive Medicine, Saarland University Hospital, Homburg, Saarland Germany; 2 Institute of Pathology, Saarland University Hospital, Homburg, Saarland Germany

**Keywords:** Bartholin gland, Vulva, Ovarian cancer, Metastasis

## Abstract

The metastasis of a gynecological malignancy to the Bartholin gland is rare. We report the case of a 62-year-old patient who had undergone extensive treatment of metastatic ovarian cancer that involved the liver, spleen, and peritoneum. She presented with painful swelling of the left vulva. Clinical and sonographic examinations showed a solid tumor in loco typico of the Bartholin gland. Surgical excision was performed. The patient died 3 months after the diagnosis of this metastasis. We performed a systematic search of PubMed, which yielded 453 entries. We selected those with at least an abstract available in English that described metastatic lesions on the Bartholin gland (*n* = 5). The review showed that a variety of primary cancers (colorectal, medullary thyroid, breast cancer, and endometrial cancers) metastasize to this location. Some patients showed signs of visceral metastasis. Bartholin gland metastases appeared as initial and metachronous manifestations. Most patients were symptomatic, with painful swelling or abscess. Genetic alterations were mentioned in some cases. The main pathways of metastasis discussed were lymphatic, but the mechanism of such metastasis remains unclear. Surgical resection was the preferred treatment option. The literature review indicated that Bartholin gland metastasis of ovarian cancer is rare and associated with poor prognosis. Oncological reasons for vulvar pathologies should be taken into consideration in patients with metastases.

## Introduction

Ovarian cancer is known to secondarily involve the genital tract, but metastasis to the vulva is rare, accounting for 2% of all reported genital metastases [1]. Such metastatic disease most frequently involves the labium majus [2]. Another common location is the clitoris [3–9]. Vulvar metastasis has been described for gynecological malignancies of the ovaries, cervix, and endometrium, as well as for extragenital malignancies such as breast, colorectal, pancreatic, and lung cancers [3–5, 10–12]. In a series of 66 patients with vulvar metastasis, 46.9% of cases were from primary tumors of gynecological origin and 43.9% of cases were of non-gynecological origin [2]. The remaining cases were of unknown primaries [2]. Metastatic disease of the vulvar Bartholin gland is very rare [4]. Here, we report on the diagnostic assessment and treatment of Bartholin gland metastasis in a patient with ovarian cancer, with a systematic literature review.

## Case Study

### Clinical Presentation

At the end of May 2022, a 62-year-old patient with metastatic ovarian cancer presented in the Department of Gynecology, Obstetrics and Reproductive Medicine, Saarland University Hospital (Homburg, Saarland, Germany), with painful swelling of the left side of the vulva that caused problems while sitting. Four weeks previously, abscess cleavage had been performed on the left side of the patient’s vulva, and no histological feature of malignancy was observed. The patient’s ovarian cancer had been treated extensively and had metastasized to the liver, spleen, and peritoneum. On presentation, she was under palliative oral chemotherapy with treosulfan 600 mg/m^2^ d1–d28 q56d. The paracentesis of symptomatic ascites was required at monthly intervals.

### Oncological Epicrisis

The patient was diagnosed in a secondary hospital with high-grade ovarian cancer (G3, International Federation of Gynecology and Obstetrics stage IIIc) in October 2012 and underwent bilateral adnexectomy and omentectomy by laparotomy resulting in residual tumor (R2). She received three cycles of chemotherapy with carboplatin AUC 5, paclitaxel 175 mg/m^2^, and bevacizumab 15 mg/kg (d1/q21d). An interventional laparotomy performed in December 2012 still showed residual tumor, and the chemotherapy was extended to six cycles. The bevacizumab treatment was continued until February 2014.

The patient was further managed at our university hospital in July 2015 because of peritoneal progression. She received palliative chemotherapy with reinduction by means of four cycles of carboplatin AUC 5 (d1/q21d). Genetic testing showed a somatic breast cancer gene (BRCA) 1 mutation, and palliative treatment with olaparib 400 mg (twice a day) was started in November 2015, after chemotherapy.

In January 2021, the disease progression with a new liver metastasis was detected. Reinduction with three cycles of carboplatin AUC 5 (d1/q21d) was performed. The disease was found to be refractory against carboplatin, and the chemotherapy was switched to liposomal doxorubicin 50 mg/m^2^ (d1/q28d) in April 2021. In November 2021, new progression with splenic metastasis appeared and the chemotherapy regime was changed to oral treosulfan 600 mg/m^2^ (d1–d28/q56d).

### Diagnostic Assessment and Therapeutic Intervention

**Fig. 1:**
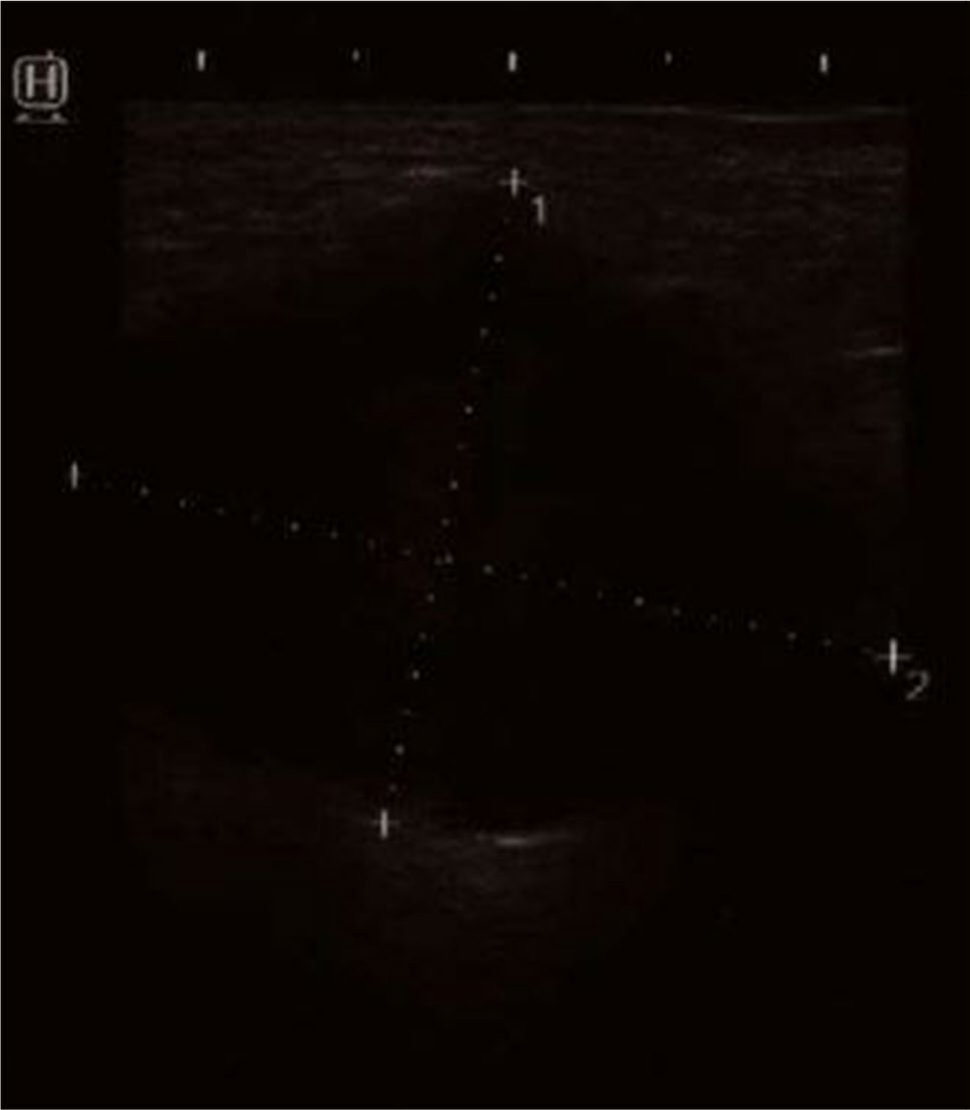
Sonographic image of the hypoechoic, solid mass of the left Bartholin gland

The patient underwent gynecological examination, which revealed a solid painful mass on the left side of the vulva in the loco typico of the Bartholin gland. Vaginal palpation revealed tumor masses on the left side of the vagina and at the apical vaginal pole and rectovaginal septum. There was no sign of bartholinitis or abscess. Sonographic examination of the introitus vulvae showed a 2.5 × 3.7 × 4.2-cm hypoechoic solid tumor (Fig. 1). Surgical excision of the tumor was performed with the patient under general anesthesia. The patient left the hospital on the day after surgery in good condition.

### Pathological Examination

**Fig. 2:**
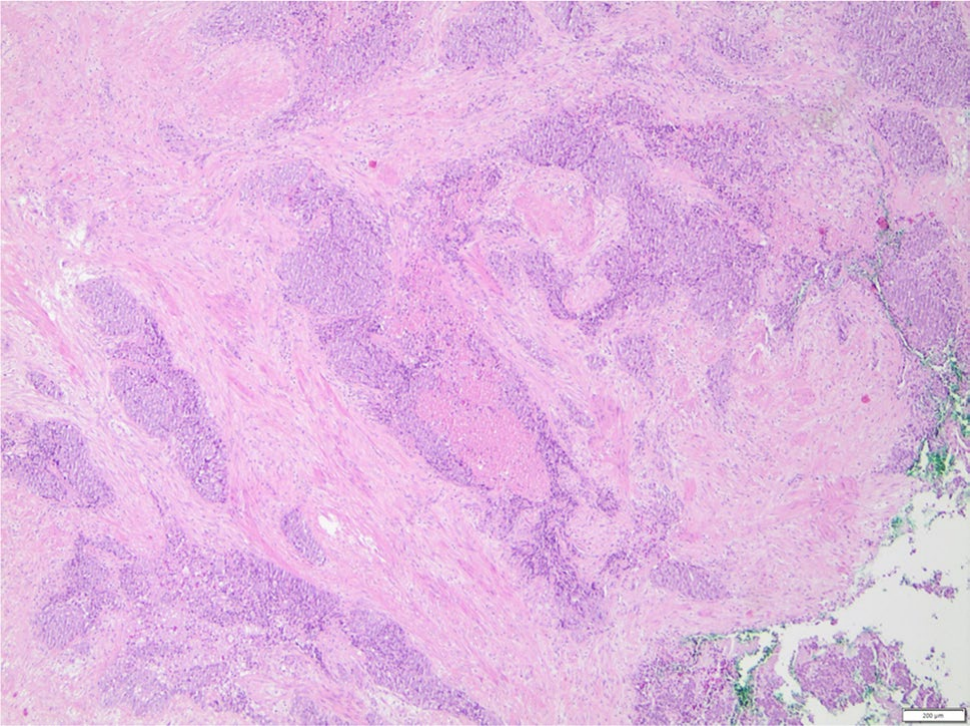
Histologic image of the Bartholin metastasis in hematoxylin eosin staining, 4 × magnification

Histological examination showed infiltration of the Bartholin gland by the metastatic high-grade serous ovarian cancer (Fig. 2). The tumor showed hemangiosis carcinomatosa and the excision was not in sano.

### Follow-Up

The patient visited the hospital’s ambulatory service for regular paracentesis. She was again able to sit without pain. The palliative treosulfan treatment was stopped after surgery, and the patient was enrolled in an outpatient palliative program. The patient died in August 2022, 3 months after the palliative excision of the Bartholin gland metastasis.

## Literature Review and Discussion

We performed a systematic literature review to compare the major features of our case with those of previously described cases. We searched PubMed using the terms “Bartholin’s gland OR Bartholin gland AND metastasis” (66 entries), “Bartholin’s gland OR Bartholin gland AND ovarian cancer” (5 entries), and “Bartholin’s gland OR Bartholin gland AND case report” (382 entries). Of the total of 453 entries, we included those with at least an abstract available in English that described metastatic lesions in the Bartholin gland; those describing metastasis in other vulvar locations, cutaneous metastasis of the vulva, primary vulvar malignancies, and lymphoma were excluded. For the included cases, data on the patient age, primary tumor, burden of disease in addition to Bartholin gland metastasis, metachronous metastasis, symptoms, genetic alterations, dissemination, and therapy were extracted.

The literature review revealed five cases of Bartholin gland metastasis [4, 13–16], but no other detailed description of such metastasis of ovarian cancer, as in our case (Table 1). Primary malignancies in the included cases were colorectal cancer, medullary thyroid carcinoma, endometrial cancer (*n* = 1 each), and breast cancer (*n* = 2). The mean patient age was 52 (range, 43 to 58) years; at 62 years of age, our patient was older.

**Table 1:** Characteristics of metastatic Bartholin tumors identified by systematic literature review

*Author*	*Year of publication*	*Patient’s age (years)*	*Primary tumor*	*Burden of disease beside the Bartholin gland*	*Metachronous metastasis*	*Symptoms*	*Genetic alteration*	*Dissemination*	*Therapy*
This case	2022	62	Ovarian cancer	Liver Spleen Peritoneum	Yes	Pain Swelling Vulva mass	Somatic BRCA 1 mutation	Hematogenous	Palliative resection
Tang et al. [13]	2018	58	Colorectal cancer	Liver	No	Ulceration Vaginal bleeding Vaginal discharge	Missense mutation G12D of K-ras	Complex	Left radical vulvectomy
Thoma-kos et al. [14]	2010	43	Medullary thyroid carcinoma	Suspicious cervical and portal vein masses	Yes, after thyroidectomy and bilateral adrenalectomy	Recurrent bartholinitis	MEN IIb syndrome	Lymphatic	Resection
Ray et al. [15]	2006	53	Endometrial cancer	No other sides	Yes, marsupialization of a benign Bartholin’s cyst curettage for postmenopausal bleeding	Vulva mass in marsupialization scar	N/A	Cell spreading and seeding	Resection
Menzin et al. [4]	1998	53	Lobular breast cancer	N/A	No	Vulva mass	N/A	N/A	Resection
Patsner [16]	1996	N/A	Breast cancer	N/A	N/A	N/A	N/A	N/A	N/A

For the case of colorectal cancer, liver metastasis is described [13]. For the case of medullary thyroid carcinoma, positron emission tomography/computed tomography showed suspicious lesions on the cervical portion of the vertebral column and intra-abdominally near the portal vein [14]. For the case of endometrial cancer, only the metastatic lesion on the Bartholin gland is described [15]. For the remaining two cases, other sites of disease burden were not mentioned in the abstract [4, 16]. In addition to the Bartholin gland lesion, our patient had metastatic lesions on the liver, spleen, and peritoneum. Comparing our case to the cases reporting other metastatic sites, these suggest that Bartholin metastasis might be associated with involvement of other organs [13, 14].

Metachronous metastasis is described for the cases of medullary thyroid carcinoma and endometrial cancer [14, 15]. The Bartholin gland lesions were the initial metastatic manifestations in one case of lobular breast cancer and the case of colorectal cancer [4, 13]. Our patient had been treated with several chemotherapeutic regimens and had metachronous dissemination. In a retrospective study of 196 patients with metastatic lesions involving the gynecological tract, 43.9% were metachronous [1].

Information about the patients’ symptoms is provided for four cases [4, 13–15]. The symptoms ranged from ulceration, vaginal bleeding, and discharge to recurrent bartholinitis and vulvar masses. In line with these findings, our patient also had a painful vulvar mass and described features of abscess on anamnesis.

Our patient had a somatic BRCA 1 mutation; genetic alterations (multiple endocrine neoplasia IIb syndrome and somatic K-Ras mutation) are mentioned in two reports included in the review [13, 14]. This could be a hint that Bartholin metastasis might be more common in patients with specific genetic alterations. The influence of genetic alterations on the Bartholin metastasis remains unclear though.

In our case, pathological examination revealed hemangiosis carcinomatosa suggesting the hematogenous dissemination of tumor cells. Dissemination to the Bartholin gland is described in three cases included in the review. In the case of endometrial cancer, the authors suspected cell spreading and seeding because the initial surgery involved diagnostic hysteroscopy with curettage and the marsupialization of a benign Bartholin’s cyst [15]. Abdullah et al. describe an isolated vulva metastasis for a patient with FIGO Ib endometrial cancer, who underwent a robotic-assisted total laparoscopic hysterectomy with vaginal specimen removal. This supports the hypothesis of malignant cell seeding [17]. The case of medullary thyroid carcinoma metastasis had extensive lymphovascular invasion, suggesting a lymphatic pathway [14]. Tang et al. [13] also discussed a lymphatic pathway of complex dissemination in the case of colorectal cancer, citing Valenzano Menada et al.’s [8] proposal of such a pathway in a case of post–plastic surgery vulvar metastasis of breast cancer. The Bartholin gland is supplied blood by the external pudendal artery, and lymphatic drainage occurs to superficial inguinal and pelvic lymph nodes [18]. Thus, the hematogenous or lymphatic dissemination of cancer cells to this gland seems to be possible.

Bartholin gland resection was performed in four cases included in the review [4, 13–15]; no treatment was reported in the fifth case [16]. According to the literature, the outcome of vulvar metastasis is poor [19]. Our patient died 3 months after palliative resection.

## Conclusion

The metastasis of ovarian cancer to the Bartholin gland is rare. Oncological reasons for the appearance of vulvar pathologies such as masses or bartholinitis should be taken into consideration, especially in patients with metastatic disease. The pathway of dissemination appears to be complex, with lymphatic and hematogenous components as well as iatrogenic spreading. Palliative resection should be considered in symptomatic patients.

## Data Availability

The dataset used and analyzed during the current study is available from the corresponding author on reasonable request.

## Code Availability

Waiver.
